# Acute onset of renal colic from bilateral ureterolithiasis: a case report

**DOI:** 10.4076/1757-1626-2-6354

**Published:** 2009-07-10

**Authors:** Eduardo de Paula Miranda, Diego Costa Almeida, Gustavo Pinto Ribeiro, Ariel Gustavo Scafuri

**Affiliations:** 1 Federal University of Ceará, Faculty of Medicine, Rua Aleandre Baraúna 949CEP: 60430-160 - Fortaleza/CEBrazil; 2 Federal University of Vale do São Francisco, Faculty of Medicine, Av. José de Sá ManiçobaS/N - CEP: 56304-917 - Petrolina/PEBrazil

## Abstract

We report a case of a 32-year-old man, who presented to the emergency department with severe abdominal pain, with radiation to his back. An ultrasound examination revealed mild hydronephrosis bilaterally. A non-enhanced computer tomography was then performed and showed a 9 mm hyperdense image in the left ureter topography along together with an 8-mm hyperdense image in the right ureter topography, allowing us to establish the diagnosis of bilateral ureterolithiasis. The patient was taken to the operating room in order to perform ureteroscopy for endoscopic removal of the stones.

## Introduction

Urolithiasis is a problem, which affects the humanity for centuries and is relatively common, with reported incidences of up to 12% of the world population during their lifetime [1]. Various studies estimate that 2-3% of all individuals present annually with either a sign or symptom related to urinary tract obstruction secondary to calculus impaction.

People aged from 20 to 30 are believed to have the highest incidence, especially men, who are affected three times more than women. Family history of urolithiasis is also an important risk factor, once up to 55% of individuals with recurrent stones report cases in the family [2].

The most likely mechanisms include: (i) the possible presence or abundance of substances that promote crystal and stone formation; (ii) a possible relative lack of substances to inhibit crystal formation; and (iii) a possible excessive excretion or concentration of salts in the urine, which leads to supersaturation of the crystallizing salt. Calcium stones account for 75-85% of urinary calculi. Approximately one half of calcium stones are composed of a mixture of calcium oxalate and calcium phosphate [3].

## Case presentation

A 32-year-old white man, who works as a construction worker, presented to the emergency department with severe and debilitating abdominal pain. The patient described the pain as colicky and diffuse throughout the anterior abdominal wall, with radiation to his back. The pain was exacerbated by walking and relieved with rest. He referred vomiting and low fever (37.8°C), but he denied any chills or night sweats. He had no previous history of urinary calculi, but he affirmed that his brother had a kidney stone, which passed spontaneously.

The patient had no known chronic medical conditions and was currently not taking any medications. He had no previous history of urinary calculi, but he affirmed that his brother had a kidney stone, which passed spontaneously. He denied alcohol, tobacco or any intravenous drug abuse.

On physical examination was 1.74 meters tall and weighed 69 kilos. The patient appeared in distress, which improved after parenteral analgesia. He was afebrile and his abdomen was diffusely tender to palpation.

**Figure 1.**
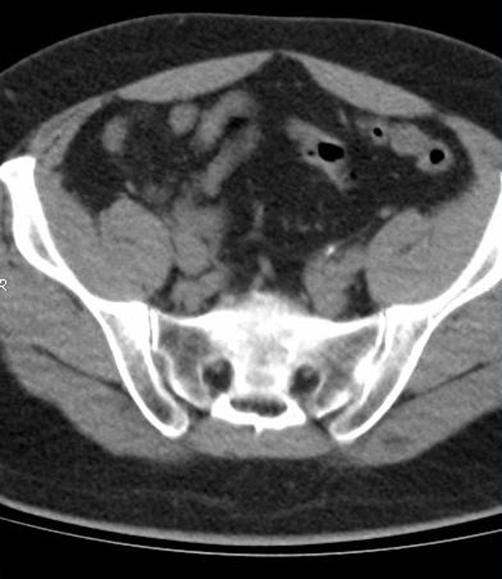
Abdominal CT revealing a 9-mm hyperdense image in the left ureter topography.

**Figure 2.**
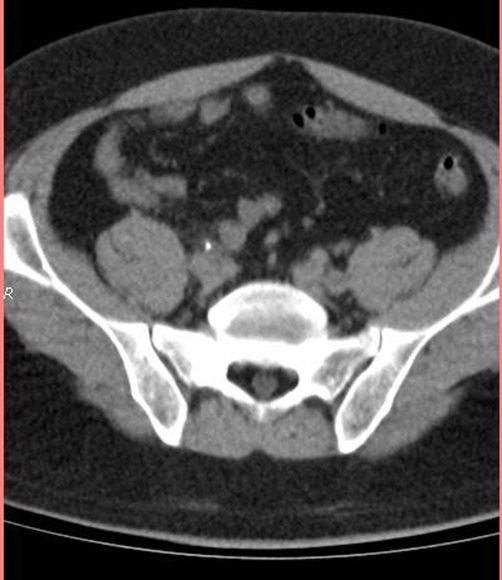
CT showing 8-mm hyperdense image in the right ureter topography.

Blood urea nitrogen and creatinine were within limits of normality; the rest of laboratorial analysis was unremarkable, except for mild leucocytosis of 12,000/μL, microhematuria, pyuria (10 leucocytes/field) and presence of crystals at urinalysis. An ultrasonography was performed and revealed mild hydronephrosis bilaterally. A nonenhanced computer tomography (CT) was then performed and showed a 9-mm hyperdense image in the left ureter topography along together with a 8-mm hyperdense image in the right ureter topography (Figures 1 and 2).The diagnosis of bilateral ureterolithiasis was then established.

The patient was taken to the operating room in order to perform ureteroscopy (URS) for removal of the stones. The procedure was uneventful and the patient was left with a double J stent at both sides to be removed at follow-up. These measures have provided immediate relief of the symptoms and 36 hours later the patient was discharged. The stone fragments were sent to analysis and the patient is currently attending a nephrologist for clinical control of idiopathic hypercalciuria.

## Discussion

Even though urolithiasis is a common affection, an acute onset of renal colic after bilateral ureterolithiasis is rather uncommon, with no similar reports in the literature.

We believe that the number of such cases is actually underestimated, probably due to spontaneous passage of the calculi before imaging tests. Besides, many other abdominal conditions may present in a similar way, leaving some cases undiagnosed.

Acute ureteral obstruction by stone usually causes severe colicky flank pain that can radiate throughout the groin, testicles, back, and periumbilical region. As the anterior abdominal pain dominated the clinical presentation of our patient, he was thought to have an acute abdominal condition, which led us to perform an abdominal ultrasonography priory to CT.

With a sensitivity of 94-97% and a specificity of 96-100%, nonenhanced helical CT is the most sensitive exam for the detection, localization, and characterization of urinary calcifications; therefore, it is considered the gold standard for approaching urinary stones. Intravenous Urography (IVU) takes more time, requires contrast and provides no additional clinically important information [4]. Thus, in places where CT is available, IVU should not be performed.

Urgent intervention by means of either percutaneous nephrostomy or ureteral stenting is indicated in a patient with an obstructed, infected upper urinary tract, rapid renal deterioration, intractable pain or vomiting, anuria, or high-grade obstruction of a solitary or transplanted kidney [2].

Distal ureteral calculi (<5 mm) usually pass the ureter spontaneously. Ureteroscopic lithotripsy of distal ureteral calculi shows high stone-free rates with a low complication rate (4%) and is equal to extracorporeal shock wave lithotripsy (ESWL), while ESWL is the primary choice for proximal ureteral stones [5]. Though the selection of these two options depends on equipments available and the expertise of the operator, URS is recommended by many authors as the optimal treatment for distal ureteral calculi [6].

The complication rate of URS is 9-11% and usually consist of avulsion of the ureteral urothelium, ureteral perforation, stricture (<1%), impaction of the instrument in the ureter with consequent ureteral laceration, extravasation of stones, and bleeding in the urogenital tract, but are minimal in experienced hands [5]. There is no evidence that bilateral approach during URS increases complication rates.

As our patient presented with 8 and 9 mm distal ureteral stone bilaterally, which is unlikely to pass spontaneously, he should be offered shock-wave lithotripsy or URS. However, our experience has led us to believe that when both ureters are affected, URS should be the procedure of choice, despite the lack of evidence in the literature.

## Abbreviations

CT: computer tomography
ESWL: extracorporeal shock wave lithotripsy
IVU: Intravenous Urography
